# Early prediction of tumor response to carbon ion radiotherapy via ^18^F-FMISO PET/CT in patients with locally advanced non-small-cell lung cancer

**DOI:** 10.3389/fonc.2025.1733152

**Published:** 2025-12-16

**Authors:** Caiyue Ren, Jian Chen, Jingfang Mao, Jiangang Zhang, Zili Li, Kailiang Wu, Jingyi Cheng

**Affiliations:** 1 Department of Nuclear Medicine, Shanghai Proton and Heavy Ion Center, Fudan University Cancer Hospital, Shanghai, China; 2 Shanghai Key Laboratory of Radiation Oncology, Shanghai, China; 3 Shanghai Engineering Research Center of Proton and Heavy Ion Radiation Therapy, Shanghai, China; 4 Department of Radiotherapy, Shanghai Proton and Heavy Ion Center, Fudan University Cancer Hospital, Shanghai, China; 5 Department of Nuclear Medicine, Shanghai Proton and Heavy Ion Center, Shanghai, China

**Keywords:** 18F-FMISO PET/CT, locally advanced non-small-cell lung cancer, carbon ion radiotherapy, response prediction, tumor hypoxia

## Abstract

**Background:**

Hypoxia increases resistance to carbon ion radiotherapy (CIRT) in locally advanced non-small-cell lung cancer (LA-NSCLC, stage II-III). This study aimed to develop a hypoxia-related model based on ^18^F-fluoromisonidazole (FMISO) positron emission tomography/computed tomography (PET/CT) for the early prediction of tumor response to CIRT in LA-NSCLC patients, thereby identifying patients most likely to benefit from CIRT.

**Methods:**

A total of 42 LA-NSCLC patients, including 25 (60%) with squamous cell carcinoma (SCC) and 17 (40%) with non-SCC, underwent ^18^F-FMISO PET/CT prior to CIRT. ^18^F-FMISO maximum standardized uptake values (SUVmax), the tumor-to-muscle ratios (TMR), and hypoxic tumor volume (HTV) were measured, with the TMR ≥ 1.4 threshold defined for hypoxia. The study endpoint was tumor response to CIRT at 3 months. After identifying ^18^F-FMISO biomarkers related to CIRT response, a prediction model was developed and evaluated using the area under curve (AUC) (95% confidence interval [CI]).

**Results:**

Among the 42 patients analyzed, 17 (40%) achieved partial response (PR) while 25 (60%) had stable disease (SD). ^18^F-FMISO-PET detected obvious uptake in 31 (74%) patients with LA-NSCLC, although these values were not related to CIRT response (*p* > 0.05). Subgroup analysis showed that favorable responses were significantly more prevalent in hypoxic SCC (TMR ≥ 1.4, n = 15, 36%) patients with smaller HTV (*p* < 0.01), whereas ^18^F-FMISO uptake was not significantly correlated with CIRT response in normoxic SCC (TMR < 1.4, n = 10, 24%) or all non-SCC (n = 17, 40%) patients (all *p* > 0.05). A positive correlation between tumor size and hypoxia was observed in SCC patients (*p* < 0.01). Multivariate logistic regression analysis showed that HTV was an independent predictor of CIRT response (*p* = 0.04, odds ratio = 1.14) with an AUC (95% CI) of 0.89 (0.71-1.00) in hypoxic SCC patients.

**Conclusion:**

^18^F-FMISO PET/CT is recommended for SCC patients, especially who are likely to have hypoxia predicted by tumor size, and it holds great promise for selecting patients who will benefit from CIRT.

## Background

Hypoxia is observed in most locally advanced non-small-cell lung cancer (LA-NSCLC, stage II-III), which holds a pivotal role in leading to tumor invasion, metastasis, and poor prognosis (1, 2). In relation to X-ray radiotherapy (RT) for LA-NSCLC patients who were unresectable or refuse surgery, hypoxia reduces the RT effectiveness and induces the radio resistance (3, 4). While dose escalation has been associated with increased locoregional control and improved overall survival, a higher X-ray radiation dose is also related with increased serious side effects, such as acute esophagitis, late lung toxicity, *etc.* (5, 6).

High-linear energy transfer (LET) carbon ion radiotherapy (CIRT) provides a new opportunity to overcome the hypoxia barrier and improve therapeutic outcome while reducing side effects due to its unique physical properties (7–9). Despite numerous *in vitro* and *in vivo* researches have demonstrated that potential clinical advantages of CIRT over X-ray RT (low-LET) for hypoxic tumors (10–12), there is less clinical evidence on the response differences to CIRT treatment in patients with highly hypoxia heterogeneous LA-NSCLC (13). Additionally, CIRT is more expensive than other treatment options, highlighting the need to identity responsive patients before treatment (14). Predicting CIRT response at an early time point would allow modification of treatment plans (e.g., dose adjustment for hypoxic areas within the tumor or alternative anti-tumor therapies for non-responsive patients), thereby improving patient prognosis (15, 16).

^18^F-fluoromisonidazole (FMISO) is the most widely used positron emission tomography (PET) tracer for mapping regional tumor hypoxia (17). ^18^F-FMISO PET/computer tomography (CT) has been confirmed the ability to characterize the distribution of hypoxia heterogeneity within tumors and gain increasing importance for its potential to predict treatment response (18, 19). The important clinical role of hypoxia imaging is to identify individuals likely to benefit from hypoxia-targeted therapy and those with poor prognosis. However, most relevant research is focused on the field of X-ray RT rather than CIRT (15, 20, 21).

Hence, this study was designed to elucidate the correlation between baseline ^18^F-FMISO-PET uptake and tumor response to CIRT in patients with LA-NSCLC. We developed and evaluated a prediction model based on ^18^F-FMISO biomarkers, which might contribute to the improvement of CIRT treatment strategies for LA-NSCLC.

## Methods

### Study design and patient recruitment

**Figure 1:**
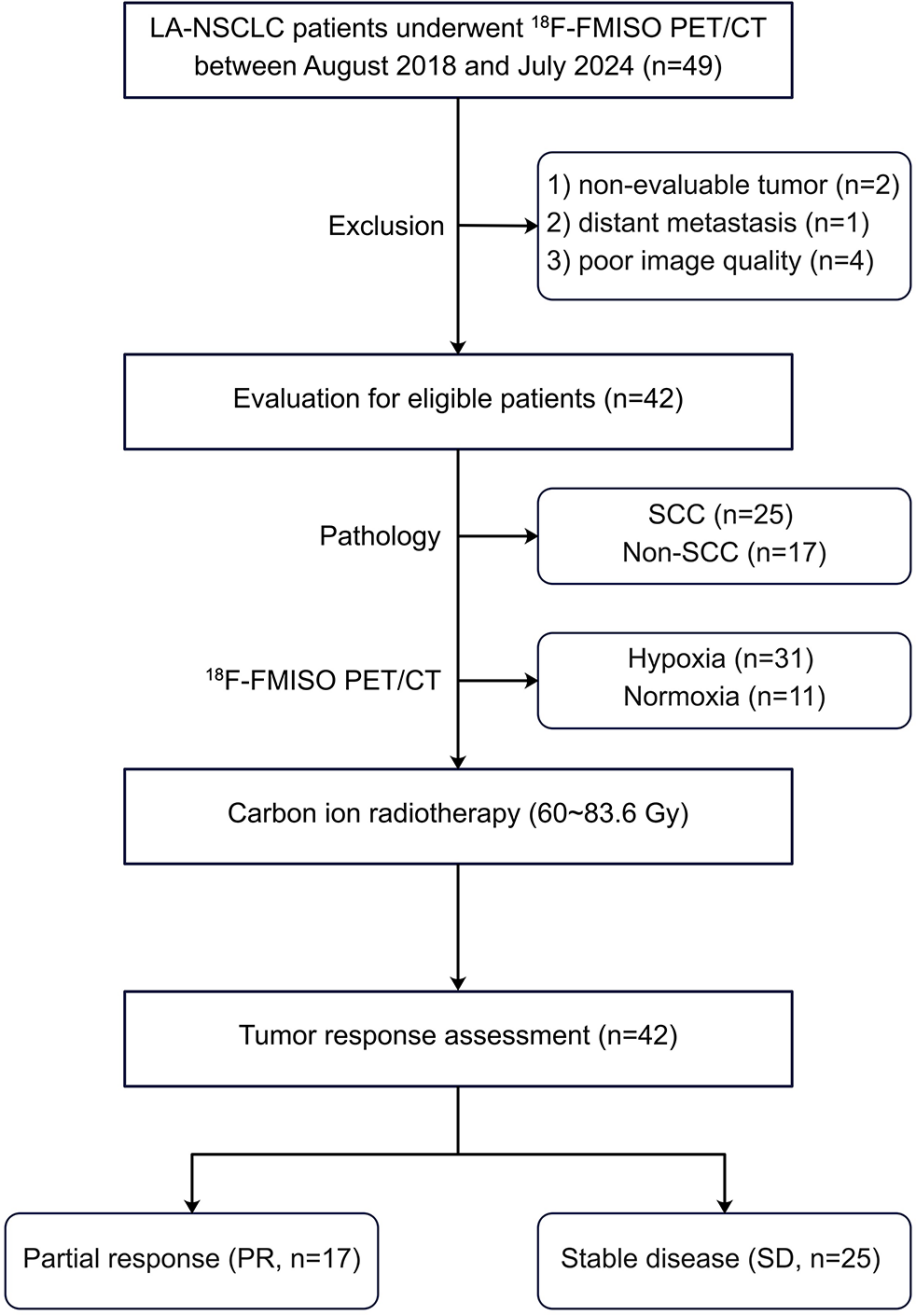
Flow chart showing the patient selection and exclusion.

The study design is described in **Figure 1**. We retrospectively reviewed the charts of LA-NSCLC patients treated with CIRT who were previously enrolled in a prospective single-center trial (Approved Number: 1707-16-03-1804A-1812B) between August 2018 and July 2024. This retrospective study was approved by the Ethics Committee of Shanghai Proton and Heavy Ion Center, and informed consent was waived.

The main inclusion criteria were as follows: 1) age ≥ 18 years; 2) histologically proven NSCLC and clinically staged II-III (8^th^ edition of the American Joint Committee on Cancer) by whole-body ^18^F-fluorodeoxyglucose (FDG) PET/CT (22); 3) thoracic ^18^F-FMISO PET/CT performed within 2 weeks before CIRT; 4) eligibility for CIRT; 5) tumor response evaluation based on thoracic contrast-enhanced CT and Response Evaluation Criteria in Solid Tumors (RECIST) 1.1 at 3 months post-CIRT (23).

The exclusion criteria included the following: 1) non-evaluable tumor before CIRT; 2) CIRT interruption due to various factors; 3) poor image quality.

### ^18^F-FMISO PET/CT protocols

The ^18^F-FMISO PET/CT scan was performed within 2 weeks before CIRT without fasting requirements, following institutional protocols detailed in our prior publication (19). A thoracic scan with 2–3 bed positions was performed on a Biograph 16 PET/CT scanner (Siemens Healthcare, Erlangen, Germany) approximately 4 hours after intravenous administering of 370 MBq of ^18^F-FMISO. Firstly, CT scans were performed (120 kVp, 150 mAs, 0.33 s per rotation, and slice thickness 3.0 mm), and CT images reconstructed (512 × 512 matrix, voxel size: 0.98 × 0.98 × 3.0 mm^3^) for attenuation correction and anatomic localization. Then, PET scans were performed with 2 min in each bed without respiratory gating, and PET images were reconstructed (200 × 200 matrix, anisotropic voxel size: 4.07 × 4.07 × 3.0 mm^3^) with the TrueX algorithm (2 iterations, 24 subsets, and 2 mm full width at half maximum).

### ^18^F-FMISO PET/CT image analysis

**Figure 2:**
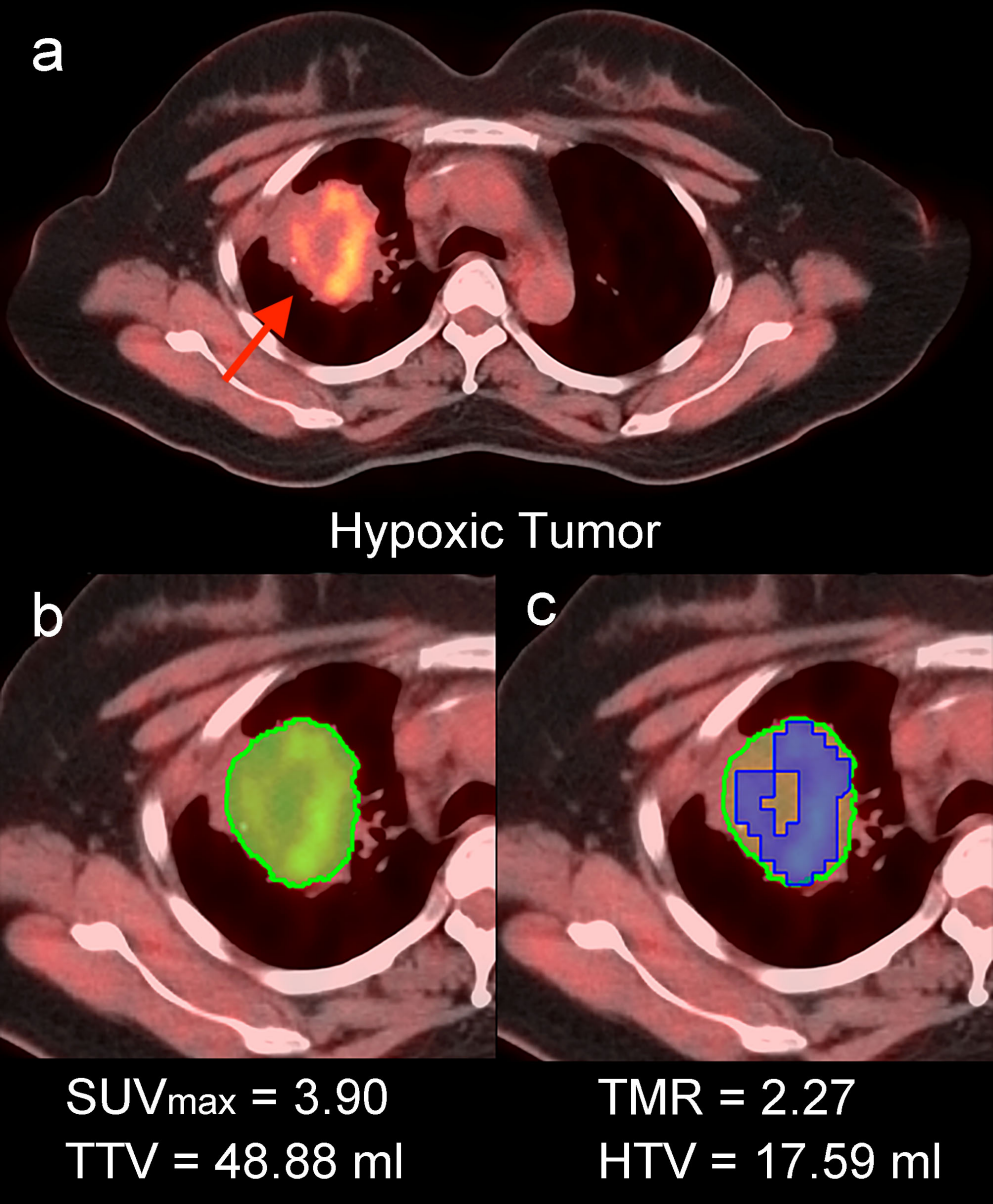
^18^F-FMISO PET/CT image analysis on the MIM software. Hypoxic tumor was defined (red arrow) by obvious ^18^F-FMISO uptake **(a)** and semi-automatedly segmented (green area) with TTV and SUVmax calculated **(b)**. The TMR was subsequently calculated **(c)** and the HTV was also defined (blue area).

Two experienced nuclear physicians independently reviewed ^18^F-FMISO PET/CT images using Medical Image Merge (MIM, version 6.5.4) software, with discrepancies resolved by consensus. The total tumor volume (TTV) was defined by the semi-automatic contouring algorithm named “PET_Edge”, which uses the maximum spatial gradient to detect boundaries between the tumor and normal tissue, free of different reconstruction algorithms, imaging techniques, and sphere diameter effects (24, 25). The maximum standardized uptake value (SUVmax) and tumor-to-muscle ratio (TMR) were calculated. The cut-off value for hypoxia was set at 1.4, and the hypoxic tumor volume (HTV) was defined as the TTV with TMR ≥1.4 according to previous studies (26, 27) (**Figure 2**).

### CIRT protocol and response evaluation

This was a purely observational study to assess the role of baseline ^18^F-FMISO PET/CT in predicting CIRT response in patients with LA-NSCLC, as all CIRT plans were independent of ^18^F-FMISO PET/CT findings. All patients with or without systemic therapies, including chemotherapy, immunotherapy, and molecular targeted therapy, prior to CIRT, but none received these treatments during the CIRT. Only primary tumors and involved lymph nodes were irradiated, and prophylactic radiation was not administered. Carbon ion beams were delivered to patients with 60-83.6 Gy/10–22 Fx using the Siemens Syngo treatment planning system (versions VC 11 & 13) (28).

Tumor response was evaluated on thoracic contrast-enhanced CT scans at 3 months post-CIRT, and only partial response (PR) and stable disease (SD) were observed in this study cohort. PR was defined as a more than 30% reduction in maximal diameter, whereas variations between −30% and +20% were classified as SD.

### Statistical analysis

All data analysis was performed on the SPSS software (version 26.0). A two-sided *p* value < 0.05 was considered statistical significance. Numerical data was represented as mean ± standard deviation and compared using independent *t-*test or Mann-Whitney U test. Categorical data was described as counts and their percentages and compared using Fisher’s exact test or χ^2^ test.

Baseline biomarkers showing the univariate relationship with CIRT response (*p* < 0.05) were entered into multivariate logistic regression model. The prediction models were developed by the linear fusion of the selected non-zero features weighted by their coefficients, with prediction scores (Pre-scores) of each model calculated for each patient. Given the small sample size of patients available in this study, the include predictors were carefully selected to ensure the simplicity and reliability of the final model. The model performance was evaluated by the receiver-operator characteristic curve (ROC) analysis, with AUC (95% confidence interval [CI]), sensitivity, specificity, and accuracy calculated. The diagnostic cut-off values were chosen by the Youden index.

## Results

### Patient characteristics and tumor hypoxia status analysis

In total, 42 LA-NSCLC patients (38 males and 4 females, mean age, 64.19 ± 10.10 years, range, 38–81 years) with baseline ^18^F-FMISO PET/CT were enrolled in this study. The most common histologic subtype was squamous cell carcinoma (SCC, n = 25, 59.5%), followed by adenocarcinoma (ADC, n = 14, 33.3%). Rarer cases of not otherwise specified (NOS) NSCLC (n = 2, 4.8%) and sarcomatoid carcinoma (n = 1, 2.4%) were reported.

^18^F-FMISO PET detected obvious uptake in 31 patients (74%) who were classified into the hypoxic groups (TMR ≥ 1.4), while slight uptake was detected in the remaining 11 patients (26%) who were classified into the normoxic groups (TMR < 1.4). The patients with hypoxic tumors had significantly higher SUVmax, TMR, and TTV than the patients with normoxic tumors (*p* < 0.05).

Tumor pathology and size (the longest diameter) demonstrated significant differences between hypoxic and normoxic groups. Specifically, hypoxia was present in about half of patients with SCC (n = 15, 60%), whereas the vast majority of patients with non-SCC (n = 16, 94%) were hypoxia (*p* = 0.01). Hypoxic tumors were generally larger than normoxic tumors (*p* < 0.01). Other baseline characteristics did not correlate with tumor hypoxia (*p* > 0.05), as shown in **Table 1**.

**Table 1:** Baseline characteristics of the 42 included locally advanced non-small cell lung cancer patients.

Characteristics	Total (n=42)	Hypoxia (n=31)	Normoxia (n=11)	*p*
Gender				
Male	38 (90.48)	28 (90.32)	10 (90.91)	0.96
Female	4 (9.52)	3 (9.68)	1 (9.09)	
Age (y)	64.19±10.10 ^*^	63.84±9.88 ^*^	65.18±11.13 ^*^	0.71
Smoking status				
Yes	34 (80.95)	24 (77.42)	10 (90.91)	0.34
No	8 (19.05)	7 (22.58)	1 (9.09)	
Tumor location				
Left lung	14 (33.33)	8 (25.81)	6 (54.55)	0.09
Right lung	28 (66.67)	23 (74.19)	5 (45.45)	
Tumor pathology				
SCC	25 (59.52)	15 (48.39)	10 (90.91)	**0.01**
Non-SCC	17 (40.48)	16 (51.61)	1 (9.09)	
Tumor size (cm)	5.90±2.30^†^	6.63±2.14^†^	3.84±1.30^†^	**<0.01**
Tumor stage (8^th^ AJCC)				
II	5 (11.90)	2 (6.45)	3 (27.27)	0.07
III	37 (88.10)	29 (93.55)	8 (72.73)	
Adjuvant therapy before CIRT				
Yes	38 (90.48)	27 (87.10)	11 (100.00)	0.22
No	4 (9.52)	4 (12.90)	0 (0.00)	
CIRT dose (Gy)	77.00 (77.00, 79.20) ^†^	77.00 (74.80, 79.20) ^†^	79.20 (77.00, 80.00) ^†^	0.12
CIRT fraction	22 (20, 22) ^†^	22 (20, 22) ^†^	22 (20, 22) ^†^	0.84
SUVmax	2.98±0.74 ^*^	3.43±0.90 ^*^	1.69±0.20 ^*^	**<0.01**
TMR	2.13±0.90 ^*^	2.45±0.59 ^*^	1.24±0.11 ^*^	**<0.01**
TTV (ml)	74.23±93.01 ^*^	94.88±100.28 ^*^	16.02±15.73 ^*^	**0.01**

SCC, squamous cell carcinoma; AJCC, American Joint Committee on Cancer; CIRT, carbon ion radiotherapy; Gy, Gray; SUVmax, maximum standardized uptake value; TMR, tumor-to-muscle ratio; TTV, total tumor volume. Data in parentheses are percentages unless otherwise noted. ^*^ Values refer to mean ± standard deviation. ^†^ Values refer to median (interquartile range). *P* values were the results of univariate analysis between hypoxic and normoxic groups, and the bold ones indicated statistical significance.

### CIRT response analysis

All patients completed CIRT treatment as planned. The tumor response was evaluated at 3 months, and 17 (40%) achieved PR while SD was observed in 25 (60%) patients. SD patients were more likely to be male smokers (*p* < 0.05), but this association was not significant in the multivariate analysis (*p* > 0.05). As presented in **Table 2**, CIRT plans, ^18^F-FMISO parameters, and other baseline characteristics exhibited no significant differences between the PR and SD groups (*p* > 0.05).

**Table 2:** Comparison of descriptive characteristics between PR and SD patients.

Characteristics	PR (n=17)	SD (n=25)	*p*
Gender			
Male	13 (76.47)	25 (100.00)	**0.01**
Female	4 (23.53)	0 (0.00)	
Age (y)	62.94±10.59 ^*^	65.04±9.89 ^*^	0.52
Smoking status			
Yes	11 (64.71)	23 (92.00)	**0.03**
No	6 (35.29)	2 (8.00)	
Tumor location			
Left lung	8 (47.06)	6 (24.00)	0.13
Right lung	9 (52.94)	19 (76.00)	
Tumor pathology			
SCC	12 (70.59)	13 (52.00)	0.24
Non-SCC	5 (29.41)	12 (48.00)	
Tumor size (cm)	5.44±1.87 ^*^	6.21±2.55 ^*^	0.30
Tumor stage (8^th^ AJCC)			
II	2 (11.76)	3 (12.00)	0.98
III	15 (88.24)	22 (88.00)	
Adjuvant therapy before CIRT			
Yes	15 (88.24)	23 (92.00)	0.69
No	2 (11.76)	2 (8.00)	
CIRT dose (Gy)	77.00 (77.00, 79.60) ^†^	77.00 (77.00, 79.20) ^†^	0.60
CIRT fraction	22 (21.00, 22) ^†^	22 (20, 22) ^†^	0.86
Baseline tumor hypoxia status			
Hypoxia	13 (76.47)	18 (72.00)	0.75
Normoxia	4 (23.53)	7 (28.00)	
SUVmax	2.98±1.08 ^*^	3.00±1.13 ^*^	0.98
TMR	2.07±0.67 ^*^	2.17±0.80 ^*^	0.68
TTV (ml)	46.61±47.13 ^*^	93.01±111.31 ^*^	0.11

PR, partial response; SD, stable disease. Data in parentheses are percentages unless otherwise noted. ^*^ Values refer to mean ± standard deviation. ^†^ Values refer to median (interquartile range). *P* values were the results of univariate analysis between PR and SD groups, and the bold ones indicated statistical significance.

As observed above, tumor pathology was significantly associated with tumor hypoxia, which might lead to different tumor responses to CIRT. Hence, further subgroup analyses were performed based on tumor pathology and hypoxia.

### CIRT response analysis for hypoxic SCC patients

Of the 15 SCC patients with hypoxic tumors, 8 (53%) achieved PR and 7 (47%) experienced SD. PR patients had significantly smaller HTV than SD patients (*p* = 0.01, **Table 3**). Multivariate logistic analysis showed that HTV was an independent significant predictor of CIRT response (*p* = 0.04, odds ratio [95% CI] = 1.14 [1.00-1.31]). Subsequently, a prediction model (Model 1) was developed and the corresponding Pre-score was calculated using the following formula:

**Table 3:** Comparison of ^18^F-FMISO parameters between PR and SD groups for hypoxic SCC patients.

Characteristics	PR (n=8)	SD (n=7)	*p*
SUVmax	3.47±0.94	3.75±0.81	0.55
TMR	2.44±0.58	2.90±0.43	0.11
TTV (ml)	73.06±55.08	105.58±83.99	0.39
HTV (ml)	16.34±11.14	35.42±13.38	**0.01**

HTV, hypoxic tumor volume. Data refer to mean ± standard deviation. *P* values were the results of univariate analysis between hypoxic and normoxic groups, and the bold ones indicated statistical significance.

Pre-score 1 (CIRT response for hypoxic SCC patients) = -3.40 + 0.13 * HTV (ml).

**Figure 3:**
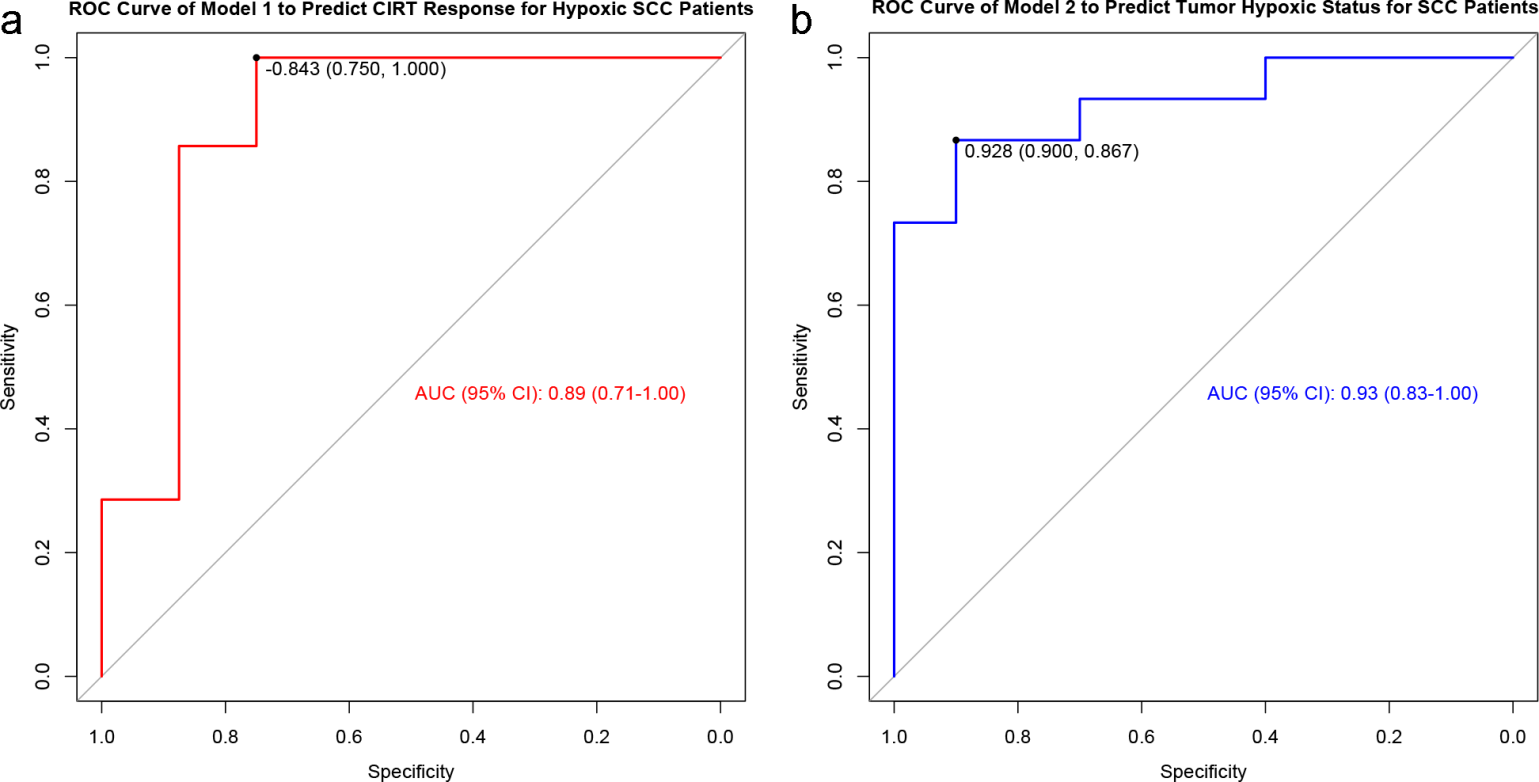
Receiver-operating characteristic analysis of models for predicting CIRT response for hypoxic SCC patients **(a)** and tumor hypoxic status for SCC patients **(b)**, respectively.

PR patients generally had lower Pre-scores than those in SD patients (*p* = 0.01). The Model 1 presented the great discrimination between PR and SD groups, with an AUC (95% CI) of 0.89 (0.71-1.00), a sensitivity of 100.0%, a specificity of 75.0%, and an accuracy of 87.7% (**Figure 3a**). The cut-off value was -0.84, and values below this threshold predict PR in patients.

As mentioned above, tumor size was positively correlated with tumor hypoxia in all LA-NSCLC patients, including those with SCC (*p* < 0.01). On this basis, an additional model (Model 2) for predicting tumor hypoxic status in SCC patients was established as follows:

Pre-score 2 (Tumor hypoxic status for SCC) = -7.32 + 1.48 * tumor size (cm).

**Figure 4:**
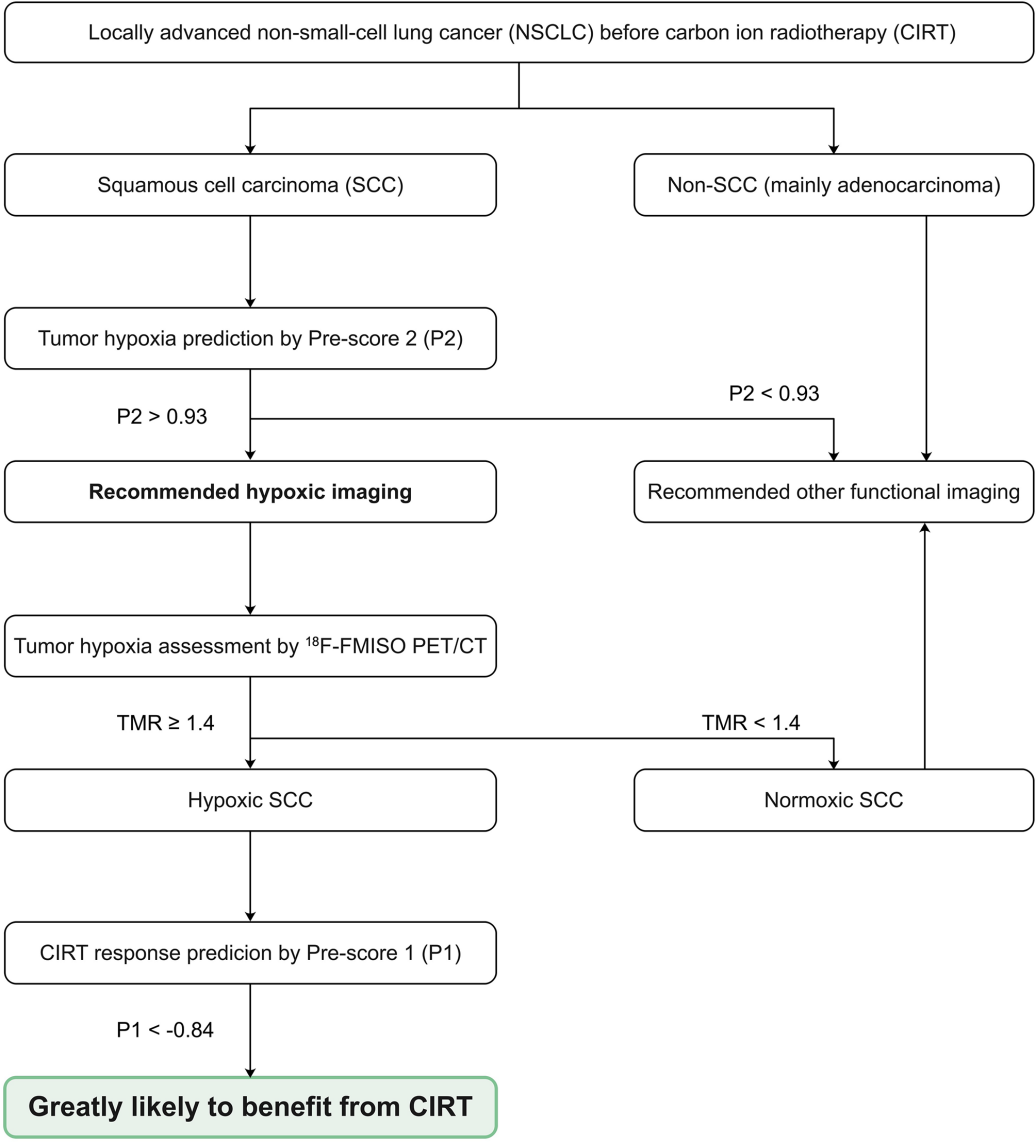
Flow chart showing the clinical applications of these two prediction models and the corresponding Pre-scores.

**Figure 5:**
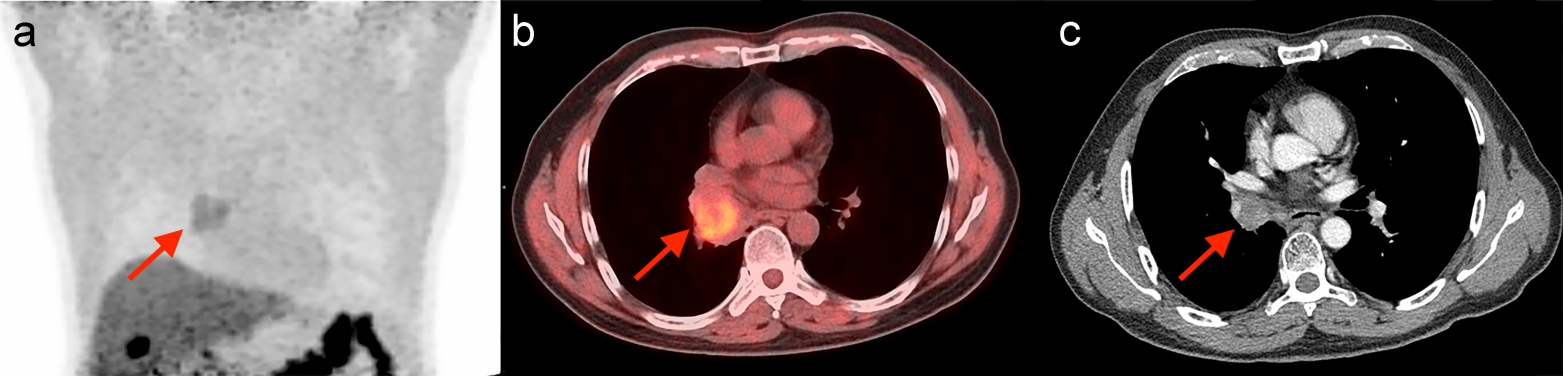
A representative case of PR prediction using the quantitative models. A 56-year-old man was pathologically diagnosed with SCC, and staged as IIIA (T4N1M0). The patient received four cycles of gemcitabine plus cisplatin chemotherapy before CIRT. The Pre-score 2 was 1.37, above the cut-off value of 0.93, and should be predicted as hypoxia. The PET/CT maximum intensity projection (MIP) **(a)** and axial fusion **(b)** images detected the single tumor (red arrow) with obvious ^18^F-FMISO uptake in the lower lobe of the right lung, with SUVmax of 3.06, TMR of 2.08, and HTV of 10.71 ml, indicating that the tumor was hypoxic. The patient received CIRT with 77 Gy/22Fx. The quantitative Pre-score 1 calculated was -1.97, below the cut-off value of -0.84, indicating a prediction of PR. Thoracic contrast-enhanced CT **(c)** after CIRT confirmed the tumor response as PR.

**Figure 6:**
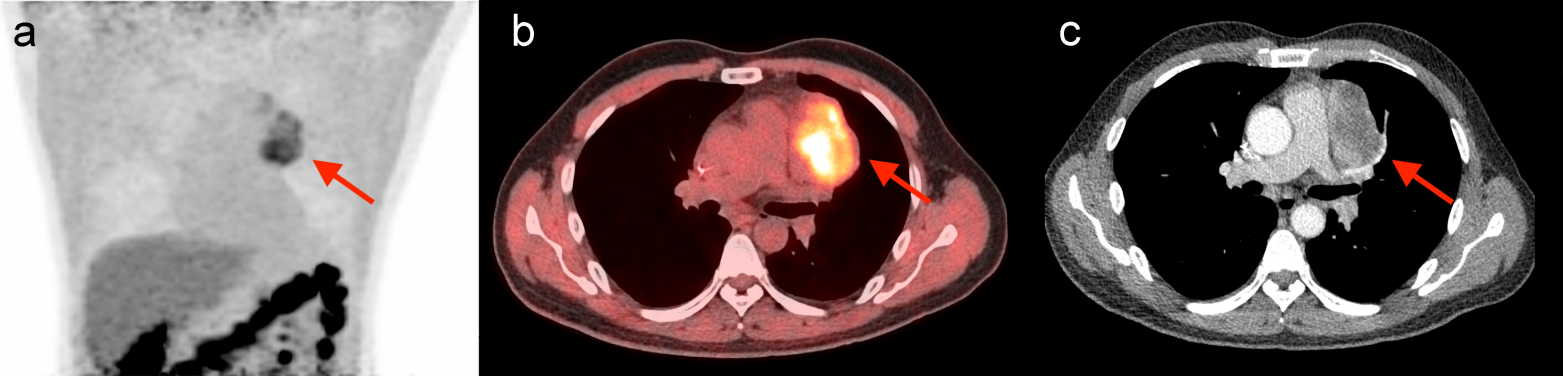
Another representative case of SD prediction. A 38-year-old man was pathologically diagnosed with SCC, and staged as IIIB (T4N2M0). The patient received six cycles of paclitaxel albumin, carboplatin, plus pembrolizumab before CIRT. The Pre-score 2 was 2.86, above the cut-off value of 0.93, and should be predicted as hypoxia. The PET/CT MIP **(a)** and axial fusion **(b)** images detected the single tumor (red arrow) with obvious ^18^F-FMISO uptake in the upper lobe of the left lung, with SUVmax of 5.26, TMR of 3.55, and HTV of 48.92 ml, indicating that the tumor was hypoxic. The patient also received CIRT with 77 Gy/22Fx. The quantitative Pre-score 1 calculated was 3.15, far above the cut-off value of -0.84, indicating a prediction of SD. Thoracic contrast-enhanced CT **(c)** after CIRT confirmed the tumor response as SD.

The Model 2 also presented the excellent discrimination between hypoxic and normoxic tumors in SCC patients, with an AUC (95% CI) of 0.93 (0.83-1.00), a sensitivity of 86.7%, a specificity of 90.0%, and an accuracy of 88.0% (**Figure 3b**). The cut-off value was 0.93, and values above this threshold predict hypoxia in SCC patients. **Figure 4** showed the clinical application flowchart for these two prediction models and the corresponding Pre-scores, while two typically cases were presented in **Figure 5** and **Figure 6**, respectively.

### CIRT response analysis for other LA-NSCLC patients

Among 10 normoxic SCC patients, 4 (40%) achieved PR and 6 (60%) experienced SD. Among 17 all non-SCC patients, 5 (29%) achieved PR and 12 (71%) experienced SD. There were no significant differences in patient’s CIRT plans and ^18^F-FMISO parameters between the PR and SD groups (*p* > 0.05, **Supplementary Table S1** in **Supplementary Data**).

## Discussion

In this study, a reliable model based on baseline ^18^F-FMISO PET/CT was developed to early predict tumor response prior to CIRT in patients with locally advanced SCC, which held an excellent performance to identify patients likely to benefit from CIRT and help clinicians optimize treatment strategies. Furthermore, an additional model was established to predict tumor hypoxic status in SCC patients, helping to identify candidates for ^18^F-FMISO PET/CT examination.

Over the past few decades, the survival rate of LA-NSCLC patients has been significantly improved due to the numerous advances in the treatment, including CIRT, but the optimal treatment has not yet been determined (29–31). Regardless of the treatment modality chosen, the prediction markers that can predict response more accurately and earlier is crucial to the next steps in individualized treatment strategies (32, 33). In our previous study (19), ΔTMR parameter, which was obtained from two ^18^F-FMISO PET/CT scans performed within 1 week before and after CIRT, showed the potential to predict treatment response with an AUC (95% CI) of 0.80 (0.61-1.00), a sensitivity of 72.7%, and an accuracy of 71.4%. As we further expanded the included sample size from 29 to 42 patients, a more accurate model consisting solely of baseline ^18^F-FMISO PET/CT parameter HTV was established to predict tumor response to CIRT with an AUC (95% CI) of 0.89 (0.71-1.00), a sensitivity of 100.0%, and an accuracy of 87.7%. The above results indicated that the ^18^F-FMISO PET/CT was useful to predict tumor response to CIRT in patients with LA-NSCLC.

In the era of novel treatment paradigm, more refined classifications contribute to the personalized medical strategies for LA-NSCLC with high heterogeneity (34, 35). Therefore, the exciting results of this study were derived from pathology- and hypoxia-based subgroup analyses rather than the overall analysis, which might be more clinically valuable. In this study, hypoxia was present in about half of SCC patients, and the multivariate analysis identified the hypoxia-related parameter HTV as a powerful predictor for predicting CIRT response for SCC patients. The negative effect of ^18^F-FMISO HTV on prognosis was observed in preclinical animal SCC models (36) and clinical oral SCC patients (37). In addition, ^18^F-FMISO has been demonstrated to be the preferred hypoxic tracer for NSCLC rather than ^18^F-Fluoroazomycin Arabinoside (27). The above results indicated that targeted therapy for tumor HTV defined by ^18^F-FMISO is expected to improve prognosis (38, 39).

Most of the included ADC patients were defined as hypoxia with obvious ^18^F-FMISO uptake, consistent with the report (40). This means the poor prognosis, as majority of them (71%) demonstrated SD after CIRT. Hayashi K, et al. reported the largest number of patients (n = 111) in evaluating the efficacy of CIRT in LA-NSCLC, and also found that ADC was a significant poor prognosticator of progression-free survival (41). With advances in targeted therapies, molecularly targeted agents can significantly improve the efficacy in patient with ADC (42, 43). Therefore, a thorough evaluation is needed to select a more appropriate treatment modality for ADC patients.

This study had several limitations. Firstly, this retrospective study included a small sample size, but it met the widely advocated sample size criterion of 10 events per variable in the development of multivariable logistic prediction model (44). Secondly, ^18^F-FMISO analysis held poor clinical value for ADC patients in the present study. A more suitable tool for those patients will be an important direction for future work. Finally, the follow-up time was not long enough. Thus, the further research will involve larger follow-up time to verify the association between findings and survival indicators such as overall survival.

In conclusion, our study has revealed the clinical significance of ^18^F-FMISO PET/CT in prediction treatment response after CIRT in LA-NSCLC patients, with quantitative prediction models established for clinical application. These findings have the potential to improve patient selection for CIRT and optimize treatment strategies.

## Data Availability

The original contributions presented in the study are included in the article/**Supplementary Material**. Further inquiries can be directed to the corresponding authors.
