# Supplementary material for: Early prediction of tumor response to carbon ion radiotherapy via 18F-FMISO PET/CT in patients with locally advanced non-small-cell lung cancer
Source: Front Oncol. 2025 Dec 16;15:1733152. doi: 10.3389/fonc.2025.1733152 (PMC12747971; doi:10.3389/fonc.2025.1733152)
Supplement: Supplementary file 1 [file Table1.docx]

Table S1 Comparison of CIRT plans and FMISO parameters between PR and SD groups for normoxic SCC and all non-SCC patients

|  | Normoxic SCC patients | | *p* | Non-SCC patients | | *p* |
| --- | --- | --- | --- | --- | --- | --- |
|  | PR (n=4) | SD (n=6) |  | PR (n=5) | SD (n=12) |  |
| CIRT dose (Gy) | 79.20 (77.55, 79.80) ^‡^ | 77.00 (77.00, 79.40) ^‡^ | 0.29 | 77.00 (68.90, 78.50) ^‡^ | 78.10 (77.00, 80.00) ^‡^ | 0.50 |
| CIRT fraction | 22.0 (20.5, 22.0) ^‡^ | 22.0 (21.5, 22.0) ^‡^ | 0.78 | 22.0 (19.0, 22.0) ^‡^ | 22.0 (20.0, 22.0) ^‡^ | 0.78 |
| Baseline tumor hypoxia status | | | - |  |  | 0.54 |
| Hypoxia | - | - |  | 5 (100.00) | 11 (91.97) |  |
| Normoxia | - | - |  | 0 (0.00) | 1 (8.33) |  |
| SUVmax | 1.67±0.14^†^ | 1.67±0.23^†^ | 0.97 | 3.26±0.91^†^ | 3.17±1.01^†^ | 0.89 |
| TMR | 1.23±0.05^†^ | 1.22±0.15^†^ | 0.91 | 2.15±0.47^†^ | 2.22±0.67^†^ | 0.86 |
| TTV | 19.24±8.58^†^ | 15.53±20.55^†^ | 0.75 | 26.18±29.12^†^ | 124.42±136.17^†^ | 0.14 |

Note: ^†^ Values refer to mean ± standard deviation. ^‡^ Values refer to median (interquartile range). *P* values were the results of univariate analysis, and the bold ones indicated statistical significance.
